# Quality of life among health care workers with and without prior COVID-19 infection in Bangladesh

**DOI:** 10.1186/s12913-022-08174-0

**Published:** 2022-06-25

**Authors:** Mahfil Ara Rahman, Soumik Kha Sagar, Koustuv Dalal, Sabrina Yesmin Barsha, Tasnim Ara, Md Abdullah Saeed Khan, Shuvajit Saha, Tanjina Sarmin, Mosharop Hossian, Mohammad Hayatun Nabi, Mohammad Lutfor Rahman, Mohammad Delwer Hossain Hawlader

**Affiliations:** 1 Department of Public Health, North South University, Dhaka, 1229 Bangladesh; 2 Department of Reproductive and Child Health, Centre for Injury Prevention & Research Bangladesh, Mohakhali, Dhaka, 1206 Bangladesh; 3 Nutrition and Clinical Services Division (NCSD), International Centre for Diarrheal Disease Research (icddr,b), Mohakhali, Dhaka, 1212 Bangladesh; 4 Division of Public Health Science, School of Health Sciences, Mid Sweden University, Sundsvall, Sweden; 5 Ibn Sina Medical College Hospital, Kallyanpur, Dhaka, 1216 Bangladesh; 6 Institute of Statistical Research and Training (ISRT), University of Dhaka, Dhaka, 1000 Bangladesh; 7 Infectious Disease Hospital, Mohakhali, Dhaka, 1212 Bangladesh; 8 Department of Maternal and Child Health, Projahnmo Research Foundation, Dhaka, Bangladesh; 9 National Institute of Preventive and Social Medicine (NIPSOM), Mohakhali, Dhaka, 1212 Bangladesh; 10 Department of Epidemiology, Public Health Professional Development Society (PPDS), Dhaka, 1205 Bangladesh

**Keywords:** Health care workers (HCWs), Quality of life (QOL), COVID-19, Bangladesh

## Abstract

**Background:**

Health care workers have been facing difficulties in coping with the COVID-19 infection from the beginning. The study aimed to compare Quality of Life (QOL) among health care workers (HCWs) with and without prior COVID-19 disease.

**Methods:**

This study was conducted from July 2020 to January 2021 among 444 HCWs. We randomly interviewed 3244 participants for our earlier nationwide survey from a list of COVID-19 positive cases after their recovery, and we found 222 HCWs among the respondents. We randomly chose 222 HCWs unaffected by COVID as a comparison group from our selected hospitals. We measured QOL using World Health Organization’s WHOQOL-BREF tool. Physical, psychological, environmental, and social ties were the four areas assessed on a 5-point Likert scale where a higher score suggests better QOL. Due to pandemic restrictions, we used telephonic interviews for data collection.

**Results:**

A higher QOL score was observed in HCWs with prior COVID-19 infection in all four domains than HCWs without previous COVID-19 conditions. Comorbidity was negatively associated with QOL scores of the physical (*p* = 0.001) and (*p* < 0.001) and psychological (*p* = 0.05, and (*p* < 0.05) domains for non-COVID and COVID-affected groups, respectively. Current smoking was significantly associated with lower psychological (*p* = 0.019) and environmental (*p* = 0.007) QOL scores among HCWs with prior COVID-19 infection. Hospitalization history due to COVID infection was a contributing factor for lower physical QOL scores (*p* = 0.048). Environmental (*p* = 0.016) QOL scores were significantly associated with the monthly income in the prior COVID-19 infection group, and physical scores were significantly associated (*p* = 0.05) with a monthly income in the non-COVID group.

**Conclusion:**

Governmental and non-governmental stakeholders should focus on potentially modifiable factors to improve health care workers’ quality of life.

**Supplementary Information:**

The online version contains supplementary material available at 10.1186/s12913-022-08174-0.

## Introduction

The world is witnessing the most critical period of the century, with the COVID-19 pandemic infecting millions of people and asserting thousands of lives. A substantial number of healthcare workers (HCWs) such as doctors, nurses, and others (laboratory technicians, healthcare helping/support staff) have contracted the disease with countless sacrifices to date [1]. Amnesty International estimated that globally at least 17,000 health care workers died from COVID-19 in the first year of the pandemic [2]. A Chinese study showed 3000 HCWs became infected (3.8%) with five deaths by early February 2020 [3]. This rate spiked to 10.5% in Italy in late April, and 157 HCW deaths were confirmed in England till early May 2020 [4–7]. In Bangladesh, among frontlines COVID-19 fighters, the highest mortality rate was observed in HCWs [8]. It has been reported that 3106 doctors, 2281 nurses, and 4015 other HCWs were infected with COVID-19 until August 28, 2021, and 186 specialized doctors died due to this viral infection illness [9]. A study by the Bangladesh Council of Scientific and Industrial Research (BCSIR) stated that the mutation rate of Coronavirus in Bangladesh was 12.6%. In comparison, the current global average is 7.23% which is very alarming [10].

HCWs are on the frontlines of this worldwide catastrophe, with the enormous task of diagnosing and treating an exponentially expanding number of acutely sick COVID-19 patients under tremendous physical and psychological stress [11, 12]. HCWs have to attend numerous medical emergencies that increase the risk of psychological upset, sometimes witnessing patients’ sufferings and dying. The quarantine period may result in prolonged separation from family members. Almost half of HCWs suffered serious psychological issues in this pandemic [13].

In Bangladesh, the number of HCWs is not sufficient. WHO estimated that 3.05 doctors and 1.07 nurses are available per 10,000 populations in Bangladesh [14]. Often, they had to do extended work in a hospital. Not all hospitals had a necessary working environment in absence of required basic medical equippments and facilities. Moreover, the number of COVID-19 positive health workers increased alarmingly, and their deaths were evident. Their family members were affected simultaneously. Despite having a stressful and extended work schedule with long duration and greater exposure for infection, HCWs had received not much attention from the empoloyer and from the society. HCWs working in hospitals and treating COVID-19 patients are constantly apprehensive of getting infected and transmitting it to their family members resulting in anxiety symptoms and impaired QOL [15]. One study noted that 7.5% HCWs indicated the need for professional psychological support [16].

The ongoing pandemic and the associated lockdown measures have affected people’s lives worldwide while HCWs are not immune to the consequences [17]. The erratic change of role from health service provider to a health care seeker might lead to stigma, adjustment issues of various intensity among the HCWs [13]. It is causing stress from individual to social levels, from economic to political status, and from national to global realm [18], affecting all domains of quality of life (QOL). Several studies have reported a higher prevalence of negative mental outcomes among the HCWs [13]. The Significant psychological impact of COVID-19 was observed among both the general population and HCWs of Bangladesh [19]. Besides, the risk of COVID-19 infection was reported to be three times higher among health workers than general people [20]. Studies in previous outbreaks, like the Ebola outbreak in Africa, observed a substantial fall in physical health and psychological QOL of HCWs [21]. Hence, we hypothesized that the QOL of COVID-19 recovered HCWs might be affected more than those who did not contract the disease. However, there is a shortage of studies focusing on the impact of COVID on the QoL of HCWs concerning their infection status. Therefore, we aimed to conduct a comparative assessment of QOL between HCWs diagnosed with COVID-19 and those who were not.

## Methods

### Study design and participants

It was a cross-sectional study. We conducted this comparative study from July 2020 to January 2021 among the two groups of health care workers (Doctor, Nurse, Laboratory Technicians, and patient helping staff such as nurse maid or ward boys). In Bangladesh, inpatients facilities have provision of patient helping staff who help the patinets different activities such as for uriniating, cleaning after defection. One group experienced with COVID-19 and another non-COVID group. The current study’s sample size was 444 (222 in each group). In our earlier nationwide study [22], we collected the list of COVID-19 positive cases from the Institute of Epidemiology and Disease Control and Research (IEDCR) which contained a list of COVID-19 positive cases from the whole country. We approached randomly to 4584 patients from the list, among them 3244 patients responded with a response rate of about 71%. Among those 3244 participants, we found 222 health care workers and considered them in this study. COVID-19 positive cases were diagnosed and confirmed by Reverse Transcription-Polymerase Chain Reaction (RT-PCR). For non-COVID respondents of this study, we selected several hospitals conveniently and collected the list of health care workers from those hospitals. We randomly chose health care workers from those lists and asked whether they have a history of COVID infection. When we found them without a history of COVID illness, we included them in this study and thus obtained 222 non-COVID participants. The study excluded pregnant women, the patients under active treatment for COVID-19, and the critically ill individual.

Considering the current pandemic situation, we performed a telephonic interview. Interviewers for data collection were assigned according to their locality to avoid the language barrier. The study used the validated Bangla translated questionnaire and asked the questions in the local language. The supervisors checked the consistency and competency of the collected data regularly during the data collection period. Moreover, the data entry team started entry and cleaning procedures alongside data collection. The data entry team checked each questionnaire to see whether appropriately filled or not, and they only selected completed questionnaires for the final analysis.

### Study instruments

We used the 26-items World Health Organization (WHO) endorsed questionnaire (short version), known as WHOQOL-BREF, for all participants in the study. The WHOQOL-BREF is a brief version of the WHOQOL-100 quality of life assessment questionnaire, validated in different languages, including Bangla.

#### Sociodemographic profile

The sociodemographic part of the questionnaire assessed information about the patient’s address, age, sex, religion, the highest level of education, occupation, marital status, and monthly income.

#### Personal history, comorbidity, and symptom profile

This section consisted of questions regarding the history of patient’s hospital admission due to COVID-19, history of smoking, comorbidities such as hypertension, diabetes, heart disease, asthma/COPD, chronic kidney disease (CKD), cancer, and a list of symptoms that might occur or persists after COVID-19 infection.

#### WHOQOL-BREF

The WHOQOL Group collaborated with 15 foreign field centers to develop the later instrument to create a QOL evaluation across cultures. The WHOQOL-BREF consists of two general items and 24 particular items that mirror the 24 aspects of WHOQOL-100. The four domains in which the 24 components are classified are physical, psychological, social interaction, and environmental. Each component is assessed on a scale of 1 to 5, with a higher score reflecting a higher quality of life. Each domain score varies from 4 to 20 and is determined by multiplying the average score of all domain facets by 4. To convert the score for 0-100 scale, each participant’s mean score was replaced by standard conversion scores laid out in detail in the WHOQOL-BREF manual [23]. Before data collection, we performed a pilot test to evaluate the competency of the questionnaire. We made the necessary modifications in research instruments based on the feedback from the pilot test. The physical health domain includes items on mobility, daily activities, functional capacity, energy, pain, and sleep. The psychological domain measures include self-image, negative thoughts, positive attitudes, self-esteem, mentality, learning ability, memory concentration, religion, and mental status. The social relationships domain contains personal relationships, social support, and sex life questions. The environmental health domain covers issues related to financial resources, safety, health, and social services, living physical environment, opportunities to acquire new skills and knowledge, recreation, general environment (noise, air pollution, etc.), and transportation. The reliability of the questionnaire was measured using Cronbach’s Alpha and validity using Pearson’s correlation coefficient.

### Statistical analysis

We conducted univariate analyses to assess differences in demographic and clinical variables and differences in WHOQOL separately for COVID affected and non-COVID health care workers using percentage distribution and student’s t-test. Moreover, bivariate analyses were conducted using an unadjusted linear regression model for all four domains of WHOQOL-BREF. QoL scores were calculated following the guideline of WHOQOL-BREF. Normality of the QoL score in different domains was checked using histogram, normal curve, Q-Q plot. Age grouping was done based on three quartiles (first quartile-28, second quartile-30, and third quartile-35). It helped us to explain the proper age distribution of the sample. Finally, we included statistically significant variables (10% level of significance) from the bivariate analyses in the multiple linear regression model. We used STATA 16 [24] for data analyses.

## Results

### Reliability and validity of the questionnaire

The scale reliability coefficient for Quality of life is 0.8570, above the minimum threshold of0.7.We calculated Pearson’s correlation coefficients to construct validity. Table 1 depicts that all four domains are strongly correlated with each other (*P* < 0.05).

**Table 1:** Pearson’s correlation coefficient for the four domains of QOL

	Physical	Psychological	Social	Environmental
Physical	1			
Psychological	0.5529*	1		
Social	0.3446*	0.3929*	1	
Environmental	0.1792*	0.1367*	0.2272*	1

*p < 0.05

### Participants’ characteristics

This comparative study included 444 healthcare professionals, 222 in each group (COVID and non-COVID). The proportion of female respondents was slightly higher (50.00%) in the non-COVID group than in the COVID group (42.34%). Married participants were higher (80.18%) in the COVID group than the non-COVID group (66.67%). In terms of education, people with graduate-level education were more common (72.97%) in the non-COVID group than the COVID group (53.60%). Similarly, people with higher income were more common in the non-COVID group than in the COVID group. COVID-infected participants were found to have more chronic diseases than the non-COVID group. We found that more than 33% of the COVID infected participants had at least one chronic illness. On the other hand, only 18% of the non-COVID participants had the same. Similarly, current and past smokers were found to be more common in the COVID group (24.78%) than in the non-COVID group (16.22%). Nearly 4 out of every 10 COVID-infected participants (39.19%) were admitted to the hospitals due to COVID (Table 2).

**Table 2:** Participants’ characteristics

Variables	Non-COVID (***n*** = 222) n (%)	COVID (***n*** = 222) n (%)	***P***-value^**a**^	Overall (***n*** = 444) n (%)
**Age (years)**^**b**^				
< 28	48 (21.62)	56 (25.23)	< 0.001^***^	104 (23.42)
28–29	53 (23.87)	30 (13.51)		83 (18.69)
30–34	95 (42.79)	44 (19.82)		139 (31.31)
35+	26 (11.71)	92 (41.44)		118 (26.58)
**Sex**				
Male	111 (50.00)	128 (57.66)	0.106	239 (53.83)
Female	111 (50.00)	94 (42.34)		205 (46.17)
**Religion**				
Muslim	201 (90.54)	177 (79.73)	0.001^**^	378 (85.14)
Non-Muslim	21 (9.46)	45 (20.27)		66 (14.86)
**Education**				
SSC/HSC	5 (2.25)	60 (27.03)	< 0.001^***^	65 (14.64)
Graduate	162 (72.97)	119 (53.60)		281 (63.29)
Post-graduate	55 (24.77)	43 (19.37)		98 (22.07)
**Monthly Income (BDT)**				
< 20,000	12 (5.88)	34 (17.44)	< 0.001^***^	46 (11.53)
20,000–40,000	57 (27.94)	72 (36.92)		129 (32.33)
40,001–60,000	57 (27.94)	34 (17.44)		91 (22.81)
60,000+	78 (38.24)	55 (28.21)		133 (33.33)
**Marital status**				
Married	148 (66.67)	178 (80.18)	0.001^**^	326 (73.42)
Single/Divorced/Widowed	74 (33.33)	44 (19.82)		118 (26.58)
**Number of chronic diseases**				
0	182 (81.98)	148 (66.67)	< 0.001^***^	330 (74.32)
1	35 (15.77)	48 (21.62)		83 (18.69)
2	5 (2.25)	9 (4.05)		14 (3.15)
3 or more	0 (0.00)	17 (7.66)		17 (3.83)
**Hospitalized due to COVID**				
No	–	135 (60.81)	–	135 (60.81)
Yes	–	87 (39.19)		87 (39.19)
**Smoking habits**				
Never	186 (83.78)	167 (75.23)	< 0.001^***^	353 (79.50)
Current	36 (16.22)	39 (17.57)		75 (16.89)
Past	0 (0.00)	16 (7.21)		16 (3.60)

^a^*P*-values were determined using chi-square tests

^b^Age grouping was done based on quartiles: 1st quartile- 28, 2nd quartile − 30 and third quartile- 35

*p < 0.05, **p < 0.01, ***p < 0.001

### Quality of life (QOL) scores between non-COVID and COVID participants

**Fig. 1:**
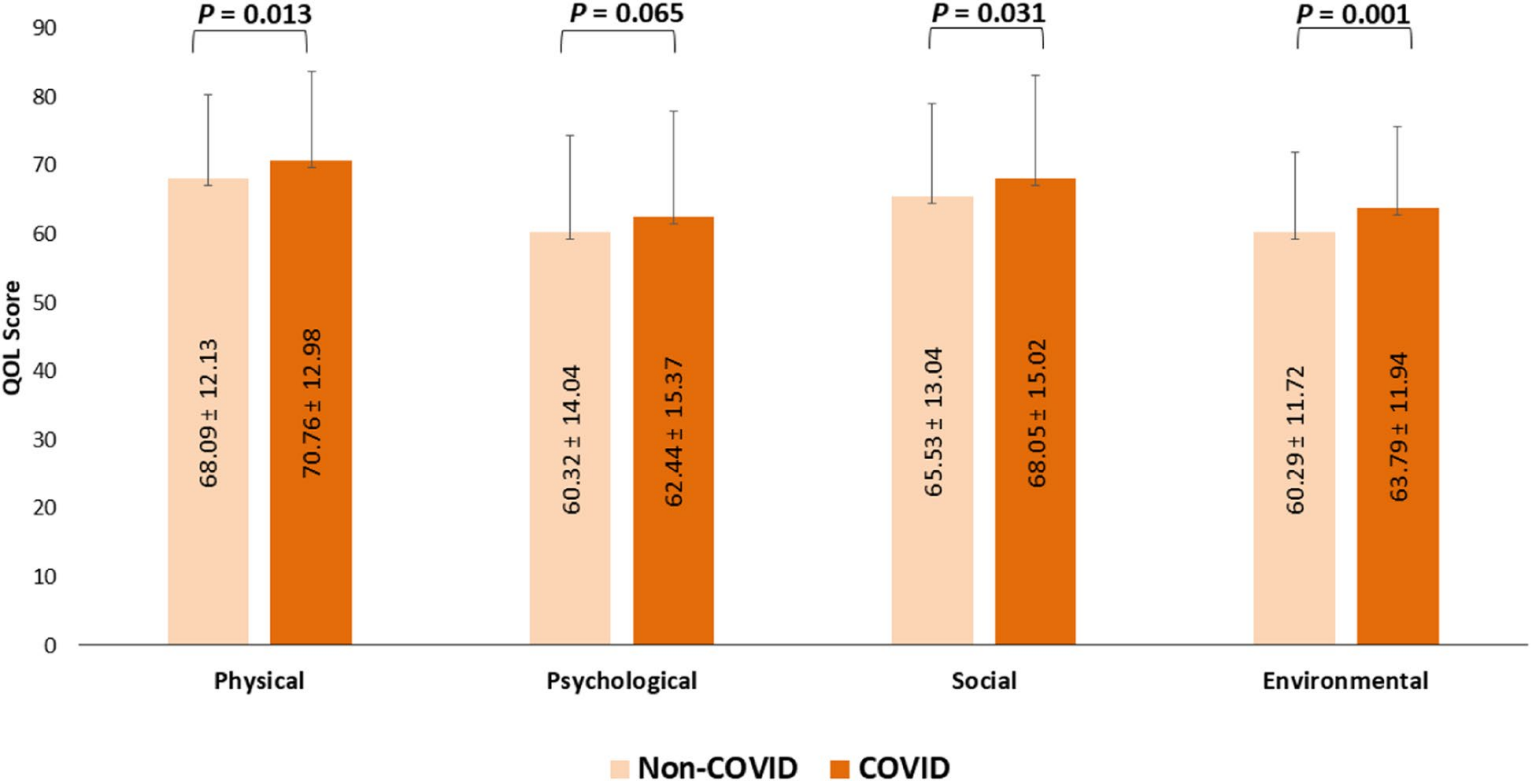
Comparison of physical, mental, social relationships, and environment QOL between Non-COVID and COVID affected health care worker

As shown in (Fig. 1), non-COVID participants had lower QOL than their COVID-infected counterparts across all four domains- physical (*p* = 0.013), psychological (*p* = 0.065), social (*p* = 0.031), and environmental (*p* = 0.001). The mean domain-specific score of health-related quality of life among the non-COVID participants was highest in the physical domain (68.09 ± 12.13), followed by the social domain (65.53 ± 13.04), psychological domain (60.32 ± 14.04), and environmental domain (60.29 ± 11.72). In COVID infected group, the mean score was highest in the physical domain (70.76 ± 12.98), followed by the social domain (68.05 ± 15.02), environmental domain (63.79 ± 11.94), and psychological domain (62.44 ± 15.37).

### Regression analysis

Linearity assumptions for univariate linear regression models for all four domains have been checked using a Quantile-quantile plot (qqnorm). The Quantile-quantile plot depicts a linear relationship between independent and dependent variables (Additional file 1). Multiple linear regression analyses were used to identify the factors associated with QOL in all four domains separately among COVID and non-COVID participants.

### Factors associated with physical QOL

After adjusting for statistically significant factors from univariate analyses, we noticed that being female (*p* = 0.001), and having a chronic disease (*p* = 0.001) were the factors that were significantly associated with a lower physical QOL among the non-COVID group. However, a monthly income of more than 40,000 had a significant positive impact on the physical QOL score (*p* < 0.05). Similar analysis within the COVID affected participants revealed that female sex (*p* = 0.008), presence of three or more chronic diseases (*p* < 0.001), hospitalization due to COVID (*p* = 0.048), and past smoking habits (*p* = 0.037) were associated with significantly lower physical QOL (Table 3).

**Table 3:** Physical domain and associated factor (results from linear regression model)

Domain	Non-COVID				COVID			
Variables	Unadjusted model		Adjusted model		Unadjusted model		Adjusted model	
	β (95% CI)	***p***-value	β (95% CI)	***p***-value	β (95% CI)	***p***-value	β (95% CI)	***p***-value
**Age (years)**								
< 28	**Reference**		**Reference**		**Reference**		**Reference**	
28–29	4.09 (− 0.66, 8.84)	0.091	4.75 (− 0.21, 9.71)	0.060	− 2.85 (− 8.59, 2.88)	0.328	− 2.85 (− 8.30, 2.59)	0.303
30–34	0.64 (− 3.58, 4.86)	0.765	0.41 (− 4.11, 4.94)	0.857	− 2.47 (− 7.58, 2.63)	0.341	− 1.73 (− 6.60, 3.13)	0.483
35+	− 0.16 (− 5.97, 5.65)	0.956	0.61 (− 5.50, 6.71)	0.845	− 5.76 (− 10.05, − 1.46)	0.009^**^	− 2.64 (− 7.01, 1.73)	0.234
**Sex**								
Male	**Reference**		**Reference**		**Reference**		**Reference**	
Female	− 4.75 (− 7.90, − 1.59)	0.003^**^	−5.40 (− 8.63, − 2.17)	0.001^**^	−3.51 (− 6.96, − 0.06)	0.046^*^	− 4.86 (− 8.41, − 1.31)	0.008^**^
**Religion**								
Muslim	**Reference**				**Reference**		**Reference**	
Non-Muslim	0.85 (− 4.64, 6.35)	0.760			−3.68 (−7.93, 0.57)	0.090	−2.97 (−7.03, 1.09)	0.151
**Education**								
SSC/HSC	**Reference**				**Reference**			
Graduate	8.59 (−2.25, 19.42)	0.120			−0.53 (−4.60, 3.54)	0.798		
Post-graduate	7.35 (−3.80, 18.49)	0.196			−0.74 (−5.88, 4.39)	0.776		
**Monthly Income (BDT)**								
< 20,000	**Reference**		**Reference**		**Reference**			
20,000–40,000	7.47 (− 0.031, 14.97)	0.051	6.10 (−1.30, 13.49)	0.106	0.04 (−5.39, 5.48)	0.988		
40,000–60,000	10.08 (2.58, 17.58)	0.009^**^	7.91 (0.49, 15.33)	0.037^*^	−0.18 (−6.51, 6.16)	0.956		
60,000+	8.90 (1.58, 16.23)	0.017^*^	8.27 (0.97, 15.58)	0.027^*^	−0.43 (−6.13, 5.27)	0.883		
**Marital status**								
Married	**Reference**		**Reference**		**Reference**			
Single/Divorced/Widowed	3.66 (0.29, 7.04)	0.034^*^	1.90 (−1.65, 5.45)	0.292	2.34 (− 1.96, 6.65)	0.285		
**Number of chronic diseases**								
0	**Reference**		**Reference**		**Reference**		**Reference**	
1	−7.40 (−11.71, −3.09)	0.001^**^	−7.57 (− 11.94, − 3.21)	0.001^**^	−4.94 (−8.97, −0.90)	0.017^*^	− 3.36 (− 7.46, 0.74)	0.108
2	−6.60 (− 17.19, 3.99)	0.221	−6.50 (− 16.79, 3.79)	0.214	−9.20 (− 17.55, − 0.85)	0.031^*^	− 6.79 (− 15.22, 1.65)	0.114
3 or more	–	–	–	–	− 14.55 (−20.77, − 8.32)	< 0.001^***^	− 13.28 (− 19.72, − 6.83)	< 0.001^***^
**Hospitalized due to COVID**								
No	–	–			**Reference**		**Reference**	
Yes	–	–			−5.72 (−9.17, −2.28)	0.001^**^	− 3.45 (− 6.86, − 0.04)	0.048^*^
**Smoking habits**								
Never	**Reference**				**Reference**		**Reference**	
Current	3.05 (−1.30, 7.39)	0.168			−2.08 (−6.61, 2.46)	0.368	−1.63 (− 6.36, 3.10)	0.498
Past	–	–			−6.13 (−12.80, 0.54)	0.072	−6.80 (−13.18, − 0.42)	0.037^*^

*p < 0.05, **p < 0.01, ***p < 0.001

### Factors associated with psychological QOL

The multiple linear regression analyses revealed that being female (*p* = 0.007) and the presence of one or two comorbid conditions (*p* < 0.05) were negatively associated with psychological QOL scores among non-COVID participants. Whereas among COVID participants, female gender (*p* < 0.001), being non-Muslim (*p* = 0.022), having multiple chronic diseases (*p* < 0.05), and being a current smoker (*p* = 0.019) were found to be significant negative determinants of psychological domain score (Table 4).

**Table 4:** Psychological domain and associated factor (results from linear regression model)

Domain	Non-COVID				COVID			
Variables	Unadjusted model		Adjusted model		Unadjusted model		Adjusted model	
	β (95% CI)	***p***-value	β (95% CI)	***p***-value	β (95% CI)	***p***-value	β (95% CI)	***p***-value
**Age (years)**								
< 28	**Reference**				**Reference**		**Reference**	
28–29	3.33 (−2.18, 8.85)	0.234			−2.32 (−9.06, 4.43)	0.500	−2.94 (− 9.52, 3.63)	0.378
30–34	−0.51 (− 5.41, 4.39)	0.837			−5.10 (−11.11, 0.91)	0.096^*^	−5.35 (− 11.35, 0.65)	0.080
35+	2.46 (−4.27, 9.20)	0.472			−7.75 (−12.81, −2.70)	0.003^**^	−4.78 (− 10.47, 0.91)	0.099
**Sex**								
Male	**Reference**		**Reference**		**Reference**		**Reference**	
Female	−4.85 (−8.51, −1.18)	0.010^*^	−4.94 (−8.54, − 1.35)	0.007^**^	−4.98 (−9.05, −0.91)	0.017^*^	− 7.76 (− 11.96, − 3.57)	< 0.001^***^
**Religion**								
Muslim	**Reference**				**Reference**		**Reference**	
Non-Muslim	0.90 (−5.45, 7.26)	0.780			−6.91 (−11.90, −1.93)	0.007^**^	− 5.60 (− 10.38, − 0.82)	0.022^*^
**Education**								
SSC/HSC	**Reference**				**Reference**			
Graduate	−9.05 (−21.60, 3.51)	0.157			2.24 (−2.54, 7.02)	0.356		
Post-graduate	−7.56 (−20.48, 5.35)	0.250			−2.87 (−8.90, 3.16)	0.349		
**Monthly Income (BDT)**								
< 20,000	**Reference**				**Reference**			
20,000–40,000	−0.64 (−9.55, 8.28)	0.888			2.89 (−3.33, 9.12)	0.361		
40,000–60,000	1.45 (−7.46, 10.37)	0.749			3.47 (−3.78, 10.73)	0.347		
60,000+	3.69 (−5.02, 12.39)	0.405			2.56 (−3.97, 9.08)	0.440		
**Marital status**								
Married	**Reference**				**Reference**		**Reference**	
Single/Divorced/Widowed	0.08 (−3.87, 4.03)	0.968			5.54 (0.48, 10.60)	0.032^*^	−0.006 (−5.47, 5.46)	0.998
**Number of chronic diseases**								
0	**Reference**		**Reference**		**Reference**		**Reference**	
1	−7.68 (−12.68, −2.69)	0.003**	−7.82 (−12.80, −2.83)	0.002^**^	−3.75 (−8.60, 1.09)	0.128	−1.83 (−6.65, 3.00)	0.456
2	−11.80 (−24.07, 0.46)	0.059	− 12.26 (− 24.38, −0.14)	0.047^*^	−16.32 (− 26.34, − 6.31)	0.002^**^	−12.59 (− 22.51, − 2.66)	0.013^*^
3 or more			–	–	−12.52 (− 20.00, − 5.05)	0.001^**^	−9.33 (− 16.91, − 1.74)	0.016^*^
**Hospitalized due to COVID**								
No	–	–			**Reference**		**Reference**	
Yes	–	–			−6.74 (−10.82, −2.67)	0.001^**^	− 3.68 (−7.73, 0.37)	0.075
**Smoking habits**								
Never	**Reference**				**Reference**		**Reference**	
Current	1.64 (−3.41, 6.68)	0.524			−5.52 (−10.88, − 0.16)	0.044^*^	−6.71 (− 12.28, − 1.13)	0.019^*^
Past	–	–			−1.79 (−9.68, 6.09)	0.654	−2.47 (− 9.98, 5.05)	0.518

*p < 0.05, **p < 0.01, ***p < 0.001

### Factors associated with social QOL

No factors were significant modifiers of the social QOL score of non-COVID participants in the adjusted analysis. However, within COVID infected female group (*p* = 0.002), single (unmarried/divorced/widowed) marital status (*p* = 0.007), presence of three or more chronic diseases (*p* = 0.030) were found to be statistically significantly associated factors with a lower social QOL score (Table 5).

**Table 5:** Factors associated with Social domain (results from linear regression model)

Domain	Non-COVID				COVID			
Variables	Unadjusted model		Adjusted model		Unadjusted model		Adjusted model	
	β (95% CI)	***p***-value	β (95% CI)	***p***-value	β (95% CI)	***p***-value	β (95% CI)	***p***-value
**Age (years)**								
< 28	**Reference**				**Reference**			
28–29	3.20 (− 1.93, 8.33)	0.220			− 2.43 (− 9.15, 4.28)	0.476		
30–34	0.15 (−4.45, 4.76)	0.947			2.10 (−3.87, 8.08)	0.489		
35+	1.71 (− 4.56, 7.98)	0.591			1.19 (−3.84, 6.22)	0.642		
**Sex**								
Male	**Reference**				**Reference**		**Reference**	
Female	−1.54 (−5.04, 1.95)	0.384			−5.50 (−9.47, − 1.54)	0.007^**^	−6.07 (− 9.95, −2.20)	0.002^**^
**Religion**								
Muslim	**Reference**				Reference			
Non-Muslim	2.95 (−2.96, 8.85)	0.326			−0.20 (−5.15, 4.76)	0.938		
**Education**								
SSC/HSC	**Reference**		**Reference**		**Reference**			
Graduate	10.34 (−1.28, 21.96)	0.081	9.61 (−2.01, 21.23)	0.104	1.18 (−3.50, 5.87)	0.620		
Postgraduate	12.09 (0.13, 24.06)	0.048^*^	11.00 (−1.01, 23.01)	0.072	4.36 (−1.55, 10.27)	0.147		
**Monthly Income (BDT)**								
< 20,000	**Reference**				**Reference**			
20,000–40,000	3.34 (−5.05, 11.73)	0.433			0.46 (−5.90, 6.82)	0.887		
40,000–60,000	2.88 (−5.51, 11.16)	0.500			3.05 (−4.36, 10.48)	0.417		
60,000+	4.08 (−4.11, 12.27)	0.327			0.98 (−5.70, 7.65)	0.773		
**Marital status**								
Married	**Reference**		**Reference**		**Reference**		**Reference**	
Single/Divorced/Widowed	−3.36 (−7.06, 0.34)	0.075	−2.93 (−6.66, 0.80)	0.123	−6.15 (−11.08, −1.22)	0.015^*^	−6.78 (−11.66, −1.90)	0.007^**^
**Number of chronic diseases**								
0	**Reference**				**Reference**		**Reference**	
1	−2.53 (−7.28, 2.22)	0.295			4.34 (−0.52, 9.20)	0.080	3.21 (−1.56, 7.99)	0.186
2	−7.10 (−18.75, 4.55)	0.231			−3.08 (−13.13, 6.98)	0.547	−5.41 (−15.25, 4.42)	0.279
3 or more	–	–			−6.68 (−14.18, 0.81)	0.080	−8.11 (−15.41, −0.80)	0.030^*^
**Hospitalized due to COVID**								
No	**–**	–			**Reference**			
Yes	–	–			0.11 (−3.96, 4.19)	0.956		
**Smoking habits**								
Never	**Reference**				**Reference**			
Current	−0.84 (−5.54, 3.84)	0.727			−3.10 (−8.38, 2.16)	0.247		
Past	–	–			−0.77 (8.53, 6.99)	0.845		

### Factors associated with environmental QOL

Since no factor was significantly associated with environmental QOL on univariate analysis among non-COVID participants, we did not conduct multivariable linear regresssion here. On the contrary, multiple linear regression analyses in the COVID group revealed that monthly income of more than 60,000 BDT (*p* = 0.016) and current smoking habit (*p* = 0.007) were the statistically significant positive modifiers of environmental QOL score among participants (Table 6).

**Table 6:** Factors associated with Environmental domain (results from linear regression model)

Domain	Non-COVID				COVID			
Variables	Unadjusted model		Adjusted model		Unadjusted model		Adjusted model	
	β (95% CI)	***p***-value	β (95% CI)	***p***-value	β (95% CI)	***p***-value	β (95% CI)	***p***-value
**Age (years)**								
< 28	**Reference**				**Reference**			
28–29	2.83 (−1.76, 7.43)	0.226			1.89 (−3.43, 7.21)	0.485		
30–34	−1.05 (−5.13, 3.03)	0.612			0.21 (−4.53, 4.94)	0.931		
35+	0.75 (−4.86, 6.37)	0.792			3.17 (−0.82, 7.15)	0.118		
**Sex**								
Male	**Reference**				**Reference**			
Female	−0.55 (−3.66, 2.56)	0.728			−2.60 (−5.79, 0.58)	0.109		
**Religion**								
Muslim	**Reference**				**Reference**			
Non-Muslim	−0.38 (−5.68, 4.93)	0.889			−0.60 (−4.53, 3.34)	0.765		
**Education**								
SSC/HSC	**Reference**				**Reference**		**Reference**	
Graduate	−5.81 (−16.26, 4.63)	0.274			0.81 (−2.84, 4.45)	0.664	0.12 (−3.71, 3.95)	0.949
Postgraduate	−2.69 (−13.43, 8.05)	0.622			7.17 (2.57, 11.78)	0.002^**^	2.32 (−3.58, 8.23)	0.439
**Monthly Income (BDT)**								
< 20,000	**Reference**				**Reference**		**Reference**	
20,000–40,000	−0.92 (−8.45, 6.61)	0.810			2.70 (−2.15, 7.55)	0.273	2.79 (− 2.09, 7.66)	0.261
40,000–60,000	−2.08 (−9.61, 5.45)	0.587			2.59 (−3.07, 8.24)	0.368	1.50 (−4.30, 7.30)	0.610
60,000+	1.59 (−5.76, 8.94)	0.670			6.97 (1.88, 12.05)	0.008^**^	6.52 (1.22, 11.81)	0.016^*^
**Marital status**								
Married	**Reference**				**Reference**			
Single/Divorced/Widowed	0.07 (−3.23, 3.36)	0.968			−1.81 (−5.77, 2.16)	0.370		
**Number of chronic diseases**								
0	**Reference**				**Reference**		**Reference**	
1	1.73 (−2.53, 5.99)	0.424			4.24 (0.35, 8.12)	0.033*	0.16 (−4.40, 4.71)	0.946
2	6.73 (−3.74, 17.20)	0.206			4.26 (− 3.77, 12.30)	0.297	−0.84 (−9.90, 8.23)	0.856
3 or more					2.43 (−3.56, 8.42)	0.425	−3.14 (−10.08, 3.80)	0.373
**Hospitalized due to COVID**								
No	–	–			**Reference**			
Yes	–	–			2.37 (−0.86, 5.60)	0.149		
**Smoking habits**								
Never	**Reference**				**Reference**		**Reference**	
Current	−0.75 (−4.96, 3.47)	0.727			7.04 (2.95, 11.13)	0.001^**^	6.60 (1.85, 11.34)	0.007^**^
Past	–				3.99 (−2.03, 10.00)	0.193	6.13 (−0.19, 12.45)	0.057

*p < 0.05, **p < 0.01, ***p < 0.001

## Discussion

Health care workers are the most vulnerable population to become infected with COVID-19. This pandemic had placed them in stressful conditions with increased patient loads and a high risk of exposure [25]. This study has assessed the quality of life among COVID infected and non-COVID health care workers after recovery. To our knowledge, this is one of earlier attempts to study the QOL of HCWs in LMICs such as Bangladesh.

COVID-recovered HCWs aged 35+ years were likely to have adverse QOL in physical and psychological domains as per unadjusted estimates. No such findings were observed in HCWs unaffected by COVID. Although age became nonsignificant as a determinant of QOL after adjustment of other factors, our previous worksamong COVID recovered people suggests that age is an important determinant of QOL, particularly in physical and psychological domains [22]. As aged people tend to have severe disese [26], and likely to bear post-COVID fatigue for a long time [27] the impact of age on physical QOL of COVID affected people can be explained. In addition, previous studies support the age-associated mental health impact in COVID-infected and recovered patients [22, 28]. In those studies, participants over 45 years of age were 52% less likely to enjoy good physical health than young participants and the increase of age was associated with negative mental health condition [22, 28]. The fear of death, infecting close ones, and several other factors might have played a role in this domain among the participants.

This study found that after adjusting all factors, the female sex had a significant negative association with physical and psychological domains in both groups and with the social relationship domain in the COVID infected group. Moreover, HCWs had to spend more hours in their workstation, resulting in hectic daily activities and a lack of energy and sleep. These physical burdens ultimately might lead to low self-esteem, failure to concentrate, and adverse mental health [13]. Moreover, in countries like Bangladesh, women have to take more household responsibilities than men. These might be some of the reasons behind our findings. Prior research showed that females are more likely to suffer from psychological distress and be affected by stressful conditions than males [29]. Similar results like ours were reported by others where females had a negative association with physical function, bodily pain, and the emotional score of QOL [30]. However, COVID affected HCWs were more likely to be affected by isolation from social ties explaining the negative social domain score.

Monthly income had a signficant positive influence on the physical QOL scores of participants without prior COVID-19 infection. Higher incomes ensure better living qualities by providing enough money to bear treatment expenses, the surrounding environment, and many other factors that add to good health that contribute to a better quality of life. Although income is a subjective indicator of QOL, the same observations were found in bariatric surgery where higher income had a positive effect on environtal domain of QOL [31]. However, no similar effects were found in COVID-recovered HCWs suggesting that being affected by the disease was associated with a prolonged physical weakness which could not be healed early by any means.

COVID HCWs who were single, divorced or widowed had a lower social quality of life. At the same time, no such associations were found in unaffected HCWs. These findings might hint at the social isolation of COVID affected individuals. Particularly, HCWs who were constantly at risk of getting infected were already under heavy stress and fear of spreading the disease to their family members. Therefore, getting the disease could have been the more isolating and detaching experience from their social connections leading to a decreased QOL in the social domain. Evidence gathered during the pandemic suggests that prevalence of loneliness increased during the COVID-19 period as a higher score than the cutoff value has been reported by 43% of the respondents [32]. Hence, during this challenging time when HCWs had to go through periods like isolation and quarantine, the sense of loneliness might have contributed to such findings.

Chronic illness was a significantly negative influencing factor among both COVID affected and unaffected HCWs in physical and psychological domains. Earlier studies have shown that chronic diseases are associated with decreased QOL in physical and psychological domains [33, 34], which explains our results.

As COVID-19 unaffected HCWs were less likely to suffer from disharmony in their social life and relationships and physical capacity constrains, it could have evened out the QOL scores in social relationship and environmental domain in these groups of HCWs, which might have diminished the independent impact of any factors in these two domains of QOL in this group.

Although our study revealed some differences in factors influencing QOL between COVID-19 affected and unaffected individuals, interestingly we observed an overall higher QOL score among COVID-recovered HCWs compared to unaffected ones. Several reasons might have worked in concert to produce such seemingly unintuitive findings. As the COVID-19 pandemic created an unprecedented emergency around the world, countries had to take special measures to tackle the rapid rise of COVID cases. Nearly all hospitals were partially or fully adapted to COVID management hospitals around the world. HCWs working in the frontline were given special incentives to keep them boosted.

On the other hand, those who contracted COVID and then recovered might have found a sense of relief from the stress associated with the illness and its imminent risk. Also, their physical health were going through a recovery phase, giving a heightened sense of physical wellbeing. The WHOQOL BREF instrument assesses QOL through subjective reporting, therefore, the higher QOL domain scores in COVID-recovered HCWs might reflected their overall efforts for adjustment into the post-COVID world with a renewed awareness.

Although this study contributes to a better understanding of the differences of QOL among HCWs, it was not without limitations. A comparison of quality of life between HCWs and the general population was not possible. We also were not able to assess the occupational factors as we collected data from non-COVID participants using the same questionnaire from our earlier nationwide study [22] where an in-depth exploration of occupational factors was beyond the scope. For non-COVID HCWs, we selected the hospitals at our convenience. A more systematic approach for the selection of hospitals could have provided a better scenario. We recommend further studies with large samples in the future. Moreover, the current study might have found ‘what’ has happened. However, a qualitative exploration of ‘why’ it happened may provide more in-depth information for a better understanding of the factors influencing quality of life of people during the times of COVID. Therefore, we recommend further largescale mixed-method studies in a similar context.

## Conclusions

Our present study found that the QOL of COVID-infected HCWs was better than the non-infected group. Though this sounds a little surprising, the reality might be actual. HCWs are considered the savior of humankind combating this crisis, and they stood at the frontline to fight this imminent health catastrophe even with inadequate personal protective equipment (PPE). Governmental and non-governmental stakeholders should focus on potentially modifiable factors, including additional training, organizational support, family support, adequate PPE, and mental health resources. Bangladesh government has already taken many steps to improve logistic facilities, but they must be aware of the impaired QOL of HCWs.

## Supplementary Information

**Additional file 1.** Normal QQ plot for residuals of Univariate analysis.

## Data Availability

Data is available on request from Dr. Mohammad Delwer Hossain Hawlader (mohammad.hawlader@northsouth.edu).

## Abbreviations

HCWs: Health are workers
QOL: Quality of Life
WHOQOL-BREF: World Health Organization Quality of Life Brief Version
