# Supplementary figures and images for: Quality of life among health care workers with and without prior COVID-19 infection in Bangladesh
Source: BMC Health Serv Res. 2022 Jun 25;22:823. doi: 10.1186/s12913-022-08174-0 (PMC9233781; doi:10.1186/s12913-022-08174-0)

Additional file 1: Normal QQ plot for residuals of Univariate analysis.


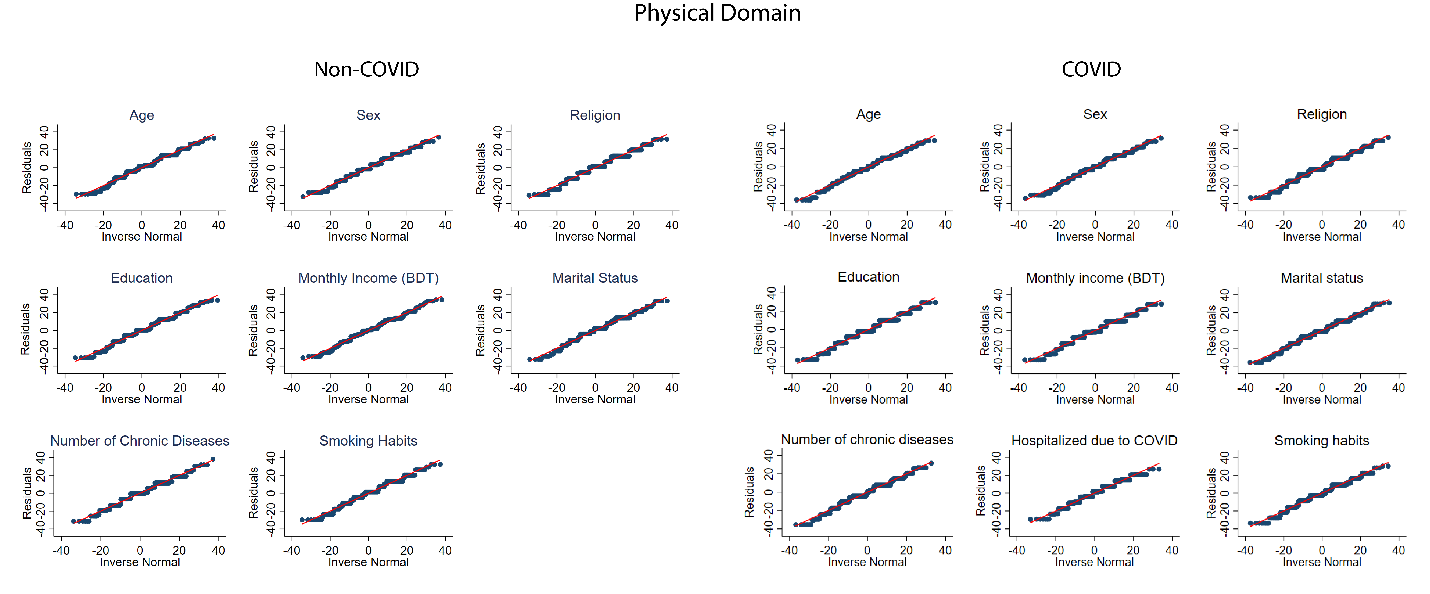


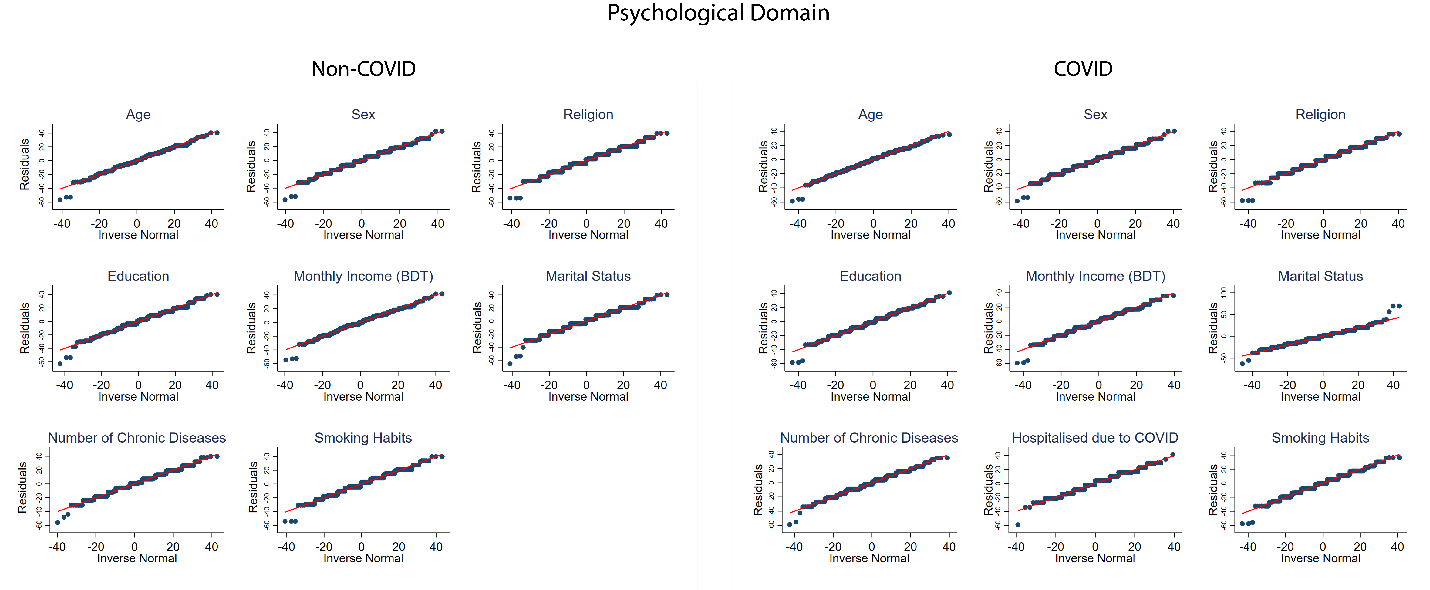


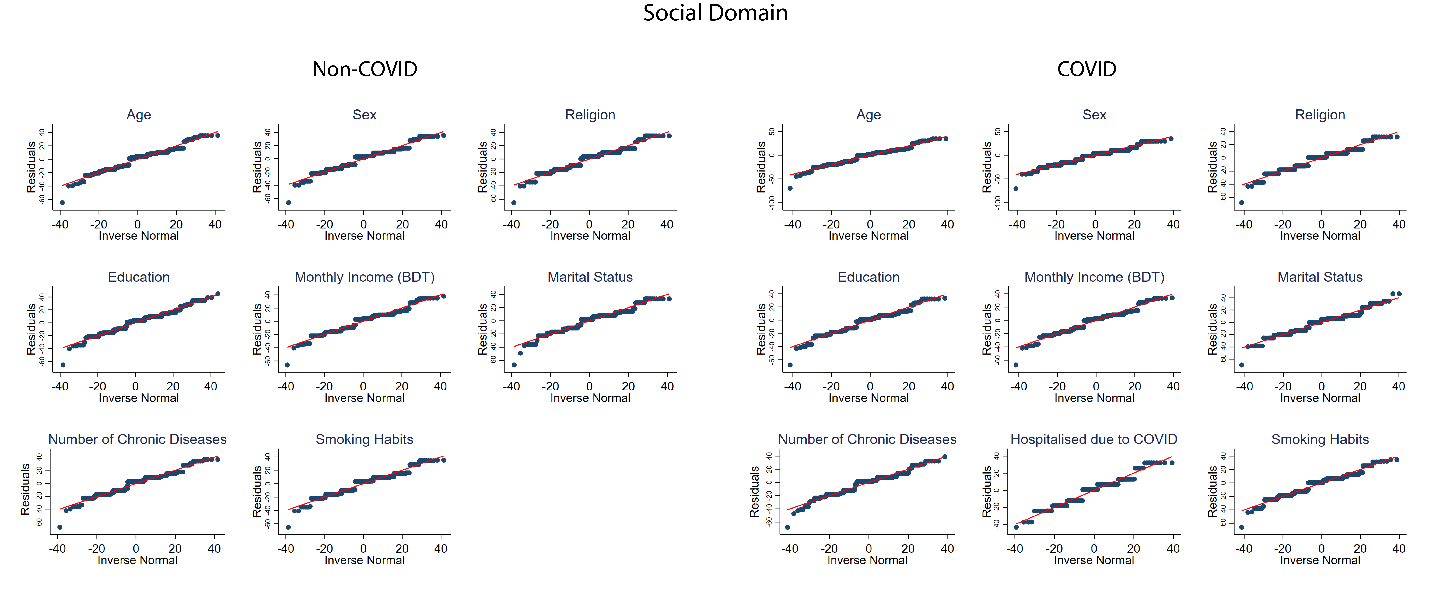


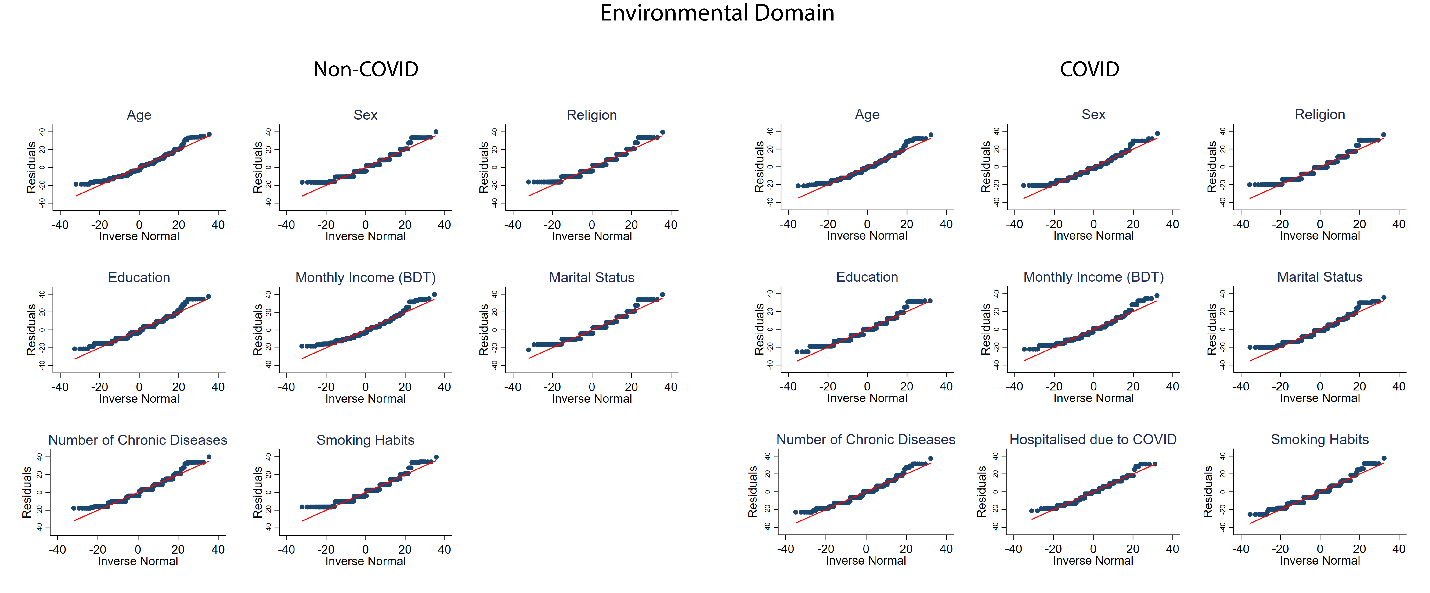

Supplement: Supplementary file 1 — Additional file 1. Normal QQ plot for residuals of Univariate analysis. [file 12913_2022_8174_MOESM1_ESM.docx]
